# ﻿Review of *Anaka* Dworakowska & Viraktamath, 1975 (Hemiptera, Cicadellidae, Typhlocybinae) with the descriptions of five new species from China

**DOI:** 10.3897/zookeys.1191.113811

**Published:** 2024-02-16

**Authors:** Meng Jiao, Maofa Yang, Xiaofei Yu, Bin Yan

**Affiliations:** 1 Institute of Entomology, Guizhou University, Guiyang, 550025, China Guizhou University Guiyang China; 2 Guizhou Provincial Key Laboratory for Agricultural Pest Management of the Mountainous Region, Guizhou University, Guiyang, 550025, China Shandong Museum Jinan China; 3 Shandong Museum, Jinan, 250014, China Guizhou University Guizhou China

**Keywords:** Auchenorrhyncha, Dikraneurini, leafhopper, morphology, taxonomy

## Abstract

The dikraneurine leafhopper genus *Anaka* is reviewed based on a comparative morphological study. Five new species, *Anakaauricula***sp. nov.**, *Anakacruciata***sp. nov.**, *Anakacurvata***sp. nov.**, *Anakarosacea***sp. nov.**, and *Anakaspiralis***sp. nov.** from China are described and illustrated in detail. Additionally, a key to known *Anaka* species is provided along with a checklist of all species and their distributions.

## ﻿Introduction

The typhlocybine leafhopper genus *Anaka* Dworakowska & Viraktamath, 1975 belonging to the tribe Dikraneurini was erected by Dworakowska and Viraktamath (1975) with *Anakacolorata* from India as the type species. Thapa and Sohi (1986) added *A.nepalica* and *A.spinosa* from Nepal. Dworakowska (1993) added *A.blada* (India), *A.sumatrana* (Indonesia), *A.shashidhari* (India), *A.burmensis* (Upper Burma), and *A.roryi* (China). Thus, eight species of the genus have been reported to date. Here, we review the previously known species and describe five new species, *Anakaauricula* sp. nov., *Anakacruciata* sp. nov., *Anakacurvata* sp. nov., *Anakarosacea* sp. nov. and *Anakaspiralis* sp. nov. from China. We also provide a key to males of all recognized species of the genus.

## ﻿Material and methods

All specimens identified in this study were collected by net trapping in southern China and are housed at the Institute of Entomology, Guizhou University, Guiyang, Guizhou, P. R. China (**GUGC:10657**).

Dry specimens were used for preparing descriptions and illustrations. External morphology was observed under a stereoscopic microscope. Body length was measured with an ocular micrometer, in millimeters, from the apex of the head to the apex of the forewing in repose. The genital segments of the specimens examined were macerated in 10% NaOH, washed in water and transferred to glycerin. Male specimens were dissected under a MOTIC B1 SMS-168 SERIES microscope. Figures were made using an OLYMPUS CX41 compound microscope. Photographs were taken with Keyence VHX-1000 and an Olympus E-520 digital camera. The digital images were then imported into Adobe Photoshop CS6 for labeling and figure composition.

## ﻿Taxonomic account

### ﻿Genus Anaka

Taxon classificationAnimaliaHemipteraCicadellidae

Dworakowska & Viraktamath, 1975

7C8111A7-4B1F-56F8-BD6E-9D4D8BF91C54

Anaka Dworakowska & Viraktamath, 1975: 521.

#### Type species.

*Anakacolorata* Dworakowska & Viraktamath, 1975 (type locality: India).

#### Description.

Vertex rounded, coronal suture distinct. Face rounded gradually to vertex, flat, lorum broad. Ocelli vestigial. Pronotum ~ 3× longer than head in dorsal view, as broad as head with eyes. Scutum with scutellum distinctly shorter than pronotum. Forewing broad, 3^rd^ apical cell stalked. Hind wing veins RP and MA confluent in male. Hindwing narrow, membrane smoked, veins dark, area bordered.

Abdominal apodemes well developed, reaching caudal margin of 4^th^ abdominal sternite. Pygofer side dark, well sclerotized, dorsal lobe the darkest, hind and ventral margins not pigmented, row of microsetae caudad. Subgenital plate has group of few big macrosetae at approximately mid-length, numerous thin short microsetae present at apical 1/3, several small rigid setae near macrosetae, and a row of thin long setae on basal 1/2 of ventral margin. Paramere hooked at apex, strongly attached to subgenital plate. Connective fused with aedeagus (Dworakowska and Viraktamath 1975). Aedeagus tubular, with basal or apical processes. Dorsoatrium well developed. Gonopore apical.

#### Distribution.

China (Guizhou, Guangdong, Guangxi, Yunnan, Sichuan, Chongqing, Hunan, Zhejiang, Fujian, Taiwan), India, Nepal, Sumatra, Indonesia, Burma.

##### ﻿Checklist of *Anaka* Dworakowska & Viraktamath, 1975

**1. *Anakaauricula* sp. nov.**

**Distribution.** China (Guizhou).

**2. *Anakablada* Dworakowska, 1993**

*Anakablada* Dworakowska, 1993a: 161.

**Distribution.** India.

**3. *Anakaburmensis* Dworakowska, 1993**

*Anakaburmensis* Dworakowska, 1993a: 163.

**Distribution.** China (Guizhou, Sichuan, Yunnan, Chongqing, Guangdong, Fujian), India.

**4. *Anakacolorata* Dworakowska & Viraktamath, 1975**

*Anakacolorata* Dworakowska & Viraktamath, 1975a: 523.

**Distribution.** India.

**5. *Anakacruciata* sp. nov.**

**Distribution.** China (Yunnan).

**6. *Anakacurvata* sp. nov.**

**Distribution.** China (Guangdong, Guangxi).

**7. *Anakanepalica* Thapa & Sohi, 1986**

*Anakanepalica* Thapa & Sohi, 1986a: 54.

**Distribution.** Nepal.

**8. *Anakaroryi* Dworakowska, 1993**

*Anakaroryi* Dworakowska, 1993c: 116.

**Distribution.** China (Taiwan).

**9. *Anakarosacea* sp. nov.**

**Distribution.** China (Guizhou).

**10. *Anakashashidhari* Dworakowska, 1993**

*Anakashashidhari* Dworakowska, 1993a: 162.

**Distribution.** India.

**11. *Anakaspinosa* Thapa & Sohi, 1986**

*Anakaspinosa* Thapa & Sohi, 1986a: 56.

**Distribution.** India, Nepal.

**12. *Anakaspiralis* sp. nov.**

**Distribution.** China (Yunnan).

**13. *Anakasumatrana* Dworakowska, 1993**

*Anakasumatrana* Dworakowska, 1993a: 162.

**Distribution.** Sumatra.

### ﻿Key to males of the genus *Anaka* Dworakowska & Viraktamath, 1975

**Table:** 

1	Aedeagus with processes basally	**2**
–	Aedeagus with processes apically	**8**
2	Aedeagal processes extended beyond apex of shaft	**3**
–	Aedeagal processes shorter than or equal to shaft	**5**
3	Aedeagal processes sculptured	**4**
–	Aedeagal processes smooth	***A.sumatrana***
4	Aedeagal processes with areolate sculpture distally and parallel grooves basally	***A.roryi***
–	Aedeagal processes with distal areolate sculpture only	***A.nepalica***
5	Aedeagal shaft with minute corrugation on ventral side	***A.shashidhari***
–	Aedeagal shaft without minute corrugation on ventral side	**6**
6	Apices of aedeagal processes twisted	***A.spiralis* sp. nov.**
–	Apices of aedeagal processes straight	**7**
7	Aedeagal stem straight, close to basal appendages	***A.burmensis***
–	Aedeagal stem curved, well separated from to basal appendages	***A.colorata***
8	Aedeagus with one pair of apical processes	**9**
–	Aedeagus with two pairs of apical processes	***A.cruciata* sp. nov.**
9	Apex of aedeagal stem not curved	**10**
–	Apex of aedeagal stem curved	**11**
10	Apices of aedeagal processes long and sculptured	***A.rosacea* sp. nov.**
–	Apices of aedeagal processes short and not sculptured	***A.spinosa***
11	Aedeagal apical processes unbranched	**12**
–	Aedeagal apical processes branched	***A.auricula* sp. nov.**
12	Aedeagal apical processes broadly curved	***A.blada***
–	Aedeagal apical processes narrowly curved	***A.curvata* sp. nov.**

### ﻿ Anaka auricula sp. nov.

Taxon classificationAnimaliaHemipteraCicadellidae

EF6486A8-F31C-5A16-8F27-746DCFE39CE9

https://zoobank.org/FFD18D4F-C13E-4AA9-9327-72711E53EE19

Fig. 1A–L

#### Type material.

***Holotype***, 1♂, China: Guizhou Province, Daozhen. 28.1892°N, 107.4294°E, H, 1700 m, 14.V.2006, collected by Yang Zaihua.

#### Description.

**Figure 1.**
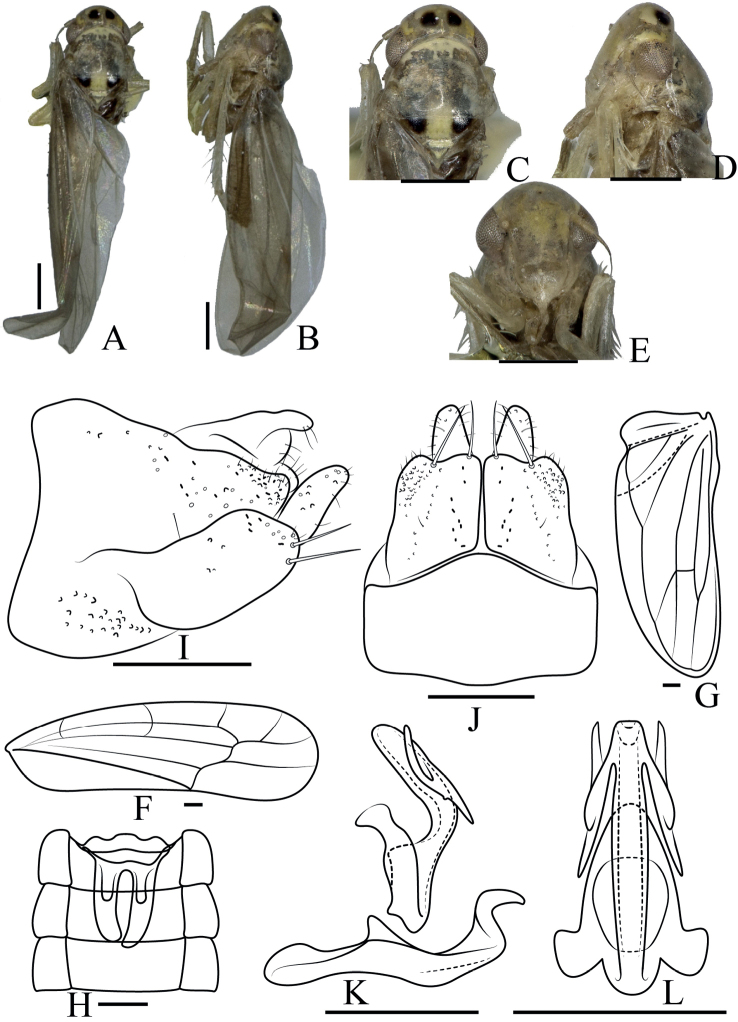
*Anakaauricula* sp. nov. **A** male body, dorsal view **B** male body, lateral view **C** head and thorax, dorsal view **D** head and thorax, lateral view **E** face **F** forewing **G** hindwing **H** abdominal apodeme **I** male pygofer, lateral view **J** subgenital plate, ventral view **K** aedeagus, connective, and paramere, lateral view **L** aedeagus and connective, ventral view. Scale bars: 0.5 mm (**A–E**); 0.1 mm (**F–L**).

***Length***: male 4.2 mm. ***Body*** (Fig. 1A, B) sandy beige. ***Crown*** (Fig. 1C) with two black patches. ***Face*** (Fig. 1D, E) yellowish, frontoclypeal area protuberant, anteclypeus broad. Pronotum yellowish brown, wider than crown. Scutellum yellowish with two blackish patches at lateral corner. ***Forewing*** (Fig. 1F) infuscate 3^rd^ apical cell stalked, hind wing (Fig. 1G) transparent.

***Male abdomen*** (Fig. 1H) well developed and reaching 4^th^ abdominal sternite. ***Pygofer side*** (Fig. 1I) broad, single row of thin setae on central and apical parts. Basal 1/2 of subgenital plate (Fig. 1J) broad, distal 1/2 slender in lateral view, two large macrosetae at approximately mid-length, several small setae near macrosetae, and scattered setae on distal 1/2. ***Paramere*** (Fig. 1K) hooked at apex. ***Aedeagus*** (Fig. 1K, L) tubular in lateral view, stem curved at middle, apical processes auricle-shaped with small spine at middle. Gonopore apical.

#### Etymology.

The specific epithet is derived from the Latin word *auricula* (an ear) referring to the shape of the aedeagal processes.

#### Remarks.

This species has an aedeagus very similar in form to that of *A.blada*, but it differs from that species in having elongated apical processes.

### ﻿ Anaka cruciata sp. nov.

Taxon classificationAnimaliaHemipteraCicadellidae

8DBC217B-13A4-5560-BEB0-9E15C9322768

https://zoobank.org/71769A36-C830-407C-8082-E4575284A965

Fig. 2A–M

#### Type material.

***Holotype***, 1♂, China: Yunnan Province, Pingbian. 22.9101°N, 103.7008°E, H, 2084 m, 22.V.2015, collected by Yan Bin.

#### Description.

**Figure 2.**
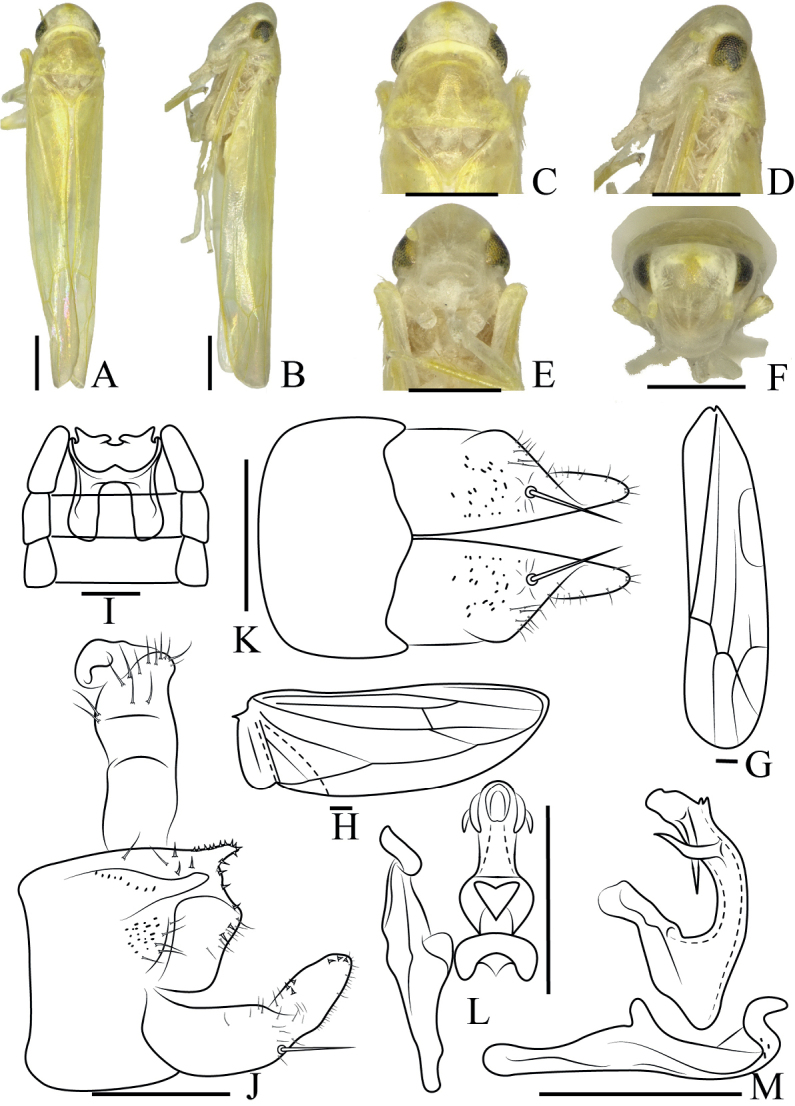
*Anakacruciata* sp. nov. **A** male body, dorsal view **B** male body, lateral view **C** head and thorax, dorsal view **D** head and thorax, lateral view **E** face **F** head, frontal view **G** forewing **H** hindwing **I** abdominal apodeme **J** male pygofer, lateral view **K** subgenital plate, ventral view **L** aedeagus, connective, and paramere, dorsal view **M** aedeagus, connective, and paramere, lateral view. Scale bars: 0.5 mm (**A–F**); 0.1 mm (**G–M**).

***Length***: male 4.2 mm. ***Body*** (Fig. 2A, B) yellowish. ***Crown*** (Fig. 2C) obtuse. Coronal suture distinct. ***Face*** (Fig. 2D–F) white, frontoclypeal area protuberant, anteclypeus broad. Pronotum yellowish, wider than crown. Scutellum small. ***Wings*** (Fig. 2G, H) without patches.

***Male abdomen*** (Fig. 2I) reaching 4^th^ abdominal sternite. ***Pygofer side*** (Fig. 2J) broad, with a small extension and thin setae on central and apical parts. Basal 1/2 of subgenital plate (Fig. 2K) broad, distal 1/2 slender in lateral view, one large macroseta at approximately midlength, several small setae near macrosetae, and scattered setae on distal 1/2. ***Paramere*** (Fig. 2L, M) hooked at apex. Connective fused with aedeagus. ***Aedeagus*** (Fig. 2L, M) tubular, curved, with two pairs of apical processes, of which each pair are crossed. Gonopore apical.

#### Etymology.

The specific epithet is derived from the Latin word *cruciatus* (marked by a cross) referring to the shape formed by the two pairs of aedeagal processes.

#### Remarks.

This species with two pairs of aedeagal processes differs from all other species of *Anaka*, and two pairs of processes originate from subapical of stem, but in different positions.

### ﻿ Anaka curvata sp. nov.

Taxon classificationAnimaliaHemipteraCicadellidae

E0369A01-A6FB-5532-96C1-88383906C096

https://zoobank.org/2B819388-B5DB-4879-948A-162E2242B86B

Fig. 3A–L

#### Type material.

***Holotype***, 1♂, China: Guangdong Province, Nanling National Natural Reserve, 24.8796°N, 113.0137°E, H, 1340 m. 4.VIII.2006, collected by Zhou Zhonghui. ***Paratypes***, 4♂♂, China: Guangxi Province, Damingshan National Natural Reserve, 23.5049°N, 108.4153°E, H, 1290 m. 15.IV.2012, collected by Long Jiankun; 6♂♂, China: Guangxi Province, Damingshan National Natural Reserve, 23.4898°N, 108.4411°E, H, 1250 m. 14.V.2012, collected by Huang Rong and Yu Xiaofei.

#### Description.

**Figure 3.**
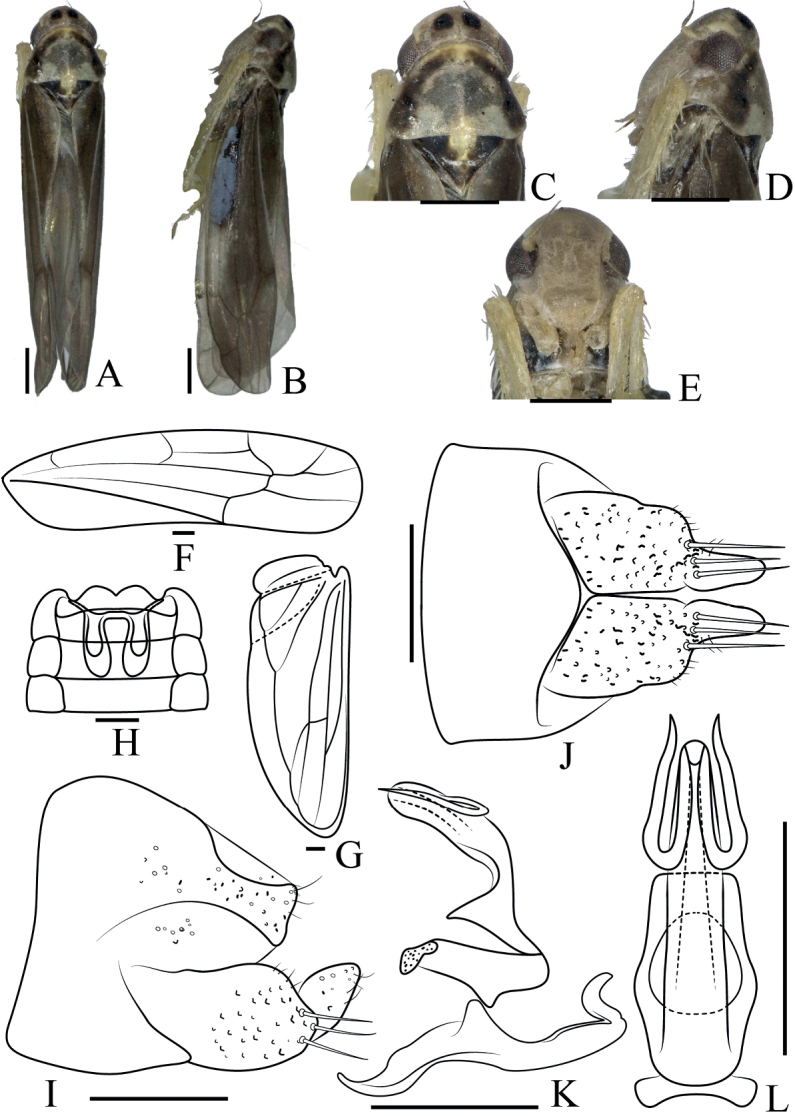
*Anakacurvata* sp. nov. **A** male body, dorsal view **B** male body, lateral view **C** head and thorax, dorsal view **D** head and thorax, lateral view **E** face **F** forewing **G** hindwing **H** abdominal apodeme **I** male pygofer, lateral view **J** subgenital plate, ventral view **K** aedeagus, connective, and paramere, lateral view **L** aedeagus, connective, ventral view. Scale bars 0.5 mm (**A–E**); 0.1 mm (**F–L**).

***Length***: male 4.4–4.5 mm. ***Body*** (Fig. 3A, B) brown. ***Crown*** (Fig. 3C) with two black patches. Coronal suture distinct. ***Face*** (Fig. 3D, E) yellowish brown, frontoclypeal area protuberant, anteclypeus broad. Pronotum brown, wider than crown. Scutellum with a vertical yellow stripe in the median. ***Forewing*** (Fig. 3F) infuscate, 3^rd^ apical cell stalked, hind wing transparent (Fig. 3G).

***Male abdomen*** (Fig. 3H) reaching 4^th^ abdominal sternite. ***Pygofer side*** (Fig. 3I) broad, thin setae on central and apical parts. Basal 1/2 of subgenital plate (Fig. 3G) broad, distal 1/2 slender in lateral view, three large macrosetae at approximately mid-length, several small setae near macrosetae, and scattered setae on distal 1/2. ***Paramere*** (Fig. 3K) hooked at apex. Connective fused with aedeagus. ***Aedeagus*** (Fig. 3K, L) tubular, curved, with a pair of apical processes, which are curved like a paper clip. Gonopore apical.

#### Etymology.

The specific epithet is derived from the Latin word *curvatus* (curved) referring to the shape of the aedeagal processes.

#### Remarks.

This species is similar to *A.blada*, but it differs in having the aedeagus processes more strongly curved and less divergent from the stem.

### ﻿ Anaka rosacea sp. nov.

Taxon classificationAnimaliaHemipteraCicadellidae

A73E0969-4B51-56F8-9931-A2155EF00F55

https://zoobank.org/43F14E58-F199-4013-86FB-2ED54327CB64

Fig. 4A–M

#### Type material.

***Holotype***, 1♂, China: Guizhou Province, Jinsha, 27.4553°N, 106.2667°E, H, 1300 m, 5.VIII.2015, collected by Zhang Yaowen. ***Paratypes***, 3♂9♀, same data as holotype.

#### Description.

**Figure 4.**
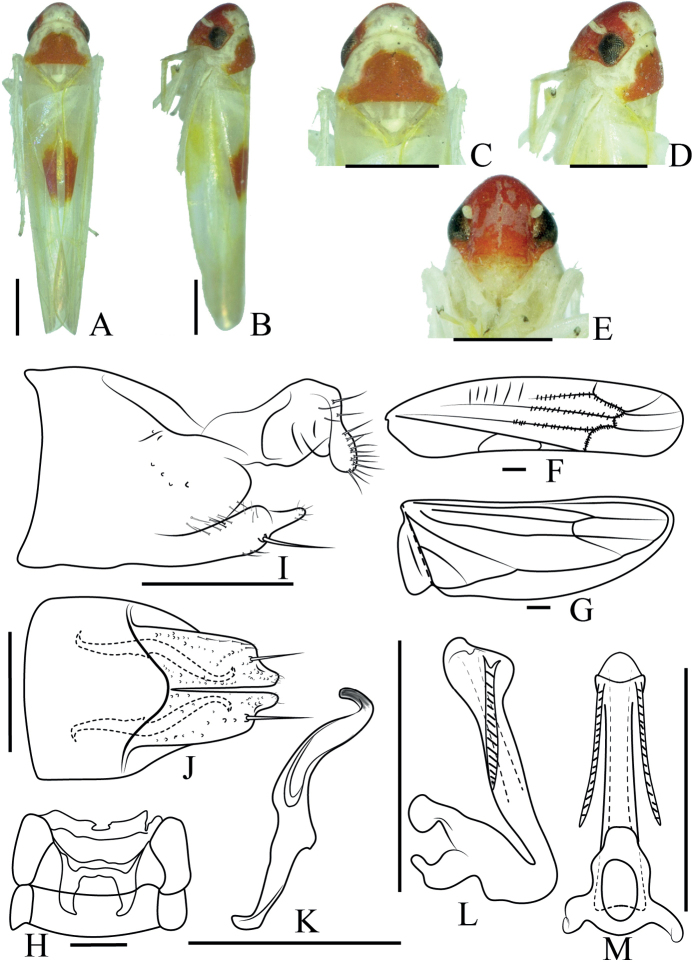
*Anakarosacea* sp. nov. **A** male body, dorsal view **B** male body, lateral view **C** head and thorax, dorsal view **D** head and thorax, lateral view **E** face **F** forewing **G** hindwing **H** abdominal apodeme **I** male pygofer, lateral view **J** subgenital plate, ventral view **K** paramere, lateral view **L** aedeagus and connective, lateral view **M** aedeagus and connective, dorsal view. Scale bars 0.5 mm (**A–E**); 0.1 mm (**F–M**).

***Length***: male 4.4–4.5 mm. ***Body*** (Fig. 4A, B) white with red patches. ***Crown*** (Fig. 4C) obtuse, yellowish white. Coronal suture distinct. ***Face*** (Fig. 4D, E) red, frontoclypeal area protuberant, anteclypeus broad, yellowish. Pronotum yellowish, with red patches in the central part, wider than crown. Scutellum yellowish. ***Forewing*** (Fig. 4F) white with red patches along inside margin, hind wing transparent (Fig. 4G).

***Male abdomen*** (Fig. 4H) weakly developed and reaching 4^th^ abdominal sternite. ***Pygofer side*** (Fig. 4I) broad, apical part elliptical. Basal 1/2 of subgenital plate (Fig. 4J) broad, distal 1/2 slender in lateral view, one large macroseta at approximately midlength. ***Paramere*** (Fig. 4K) hooked at apex. Connective fused with aedeagus. ***Aedeagus*** (Fig. 4L, M) tubular, stem inflated at apex, with one pair of apical processes, apical processes straight and sculptured, oriented basad. Gonopore apical.

#### Etymology.

The specific epithet is derived from the Latin word *rosaceus* (rose-colored) referring to the color of the head.

#### Remarks.

This species marked with rose-red spots. The aedeagal processes are similar to *A.blada* and *A.spinosa* but differs in having the aedeagus with two long apical processes and the processes straight with spiral pattern.

### ﻿ Anaka spiralis sp. nov.

Taxon classificationAnimaliaHemipteraCicadellidae

E46BA7D8-4F4D-5C3F-A799-F15C9A8B26C7

https://zoobank.org/FAB85DAA-EBFE-4621-ADED-BBB32FFD5514

Fig. 5A–N

#### Type material.

***Holotype***, 1♂, China: Yunnan Province, Baoshan, 25.1581°N, 99.0814°E, H, 1500 m, 14.V.2016, collected by Li Bin and Ren Guoru. ***Paratypes***, 3♂4♀, same data as holotype.

#### Description.

**Figure 5.**
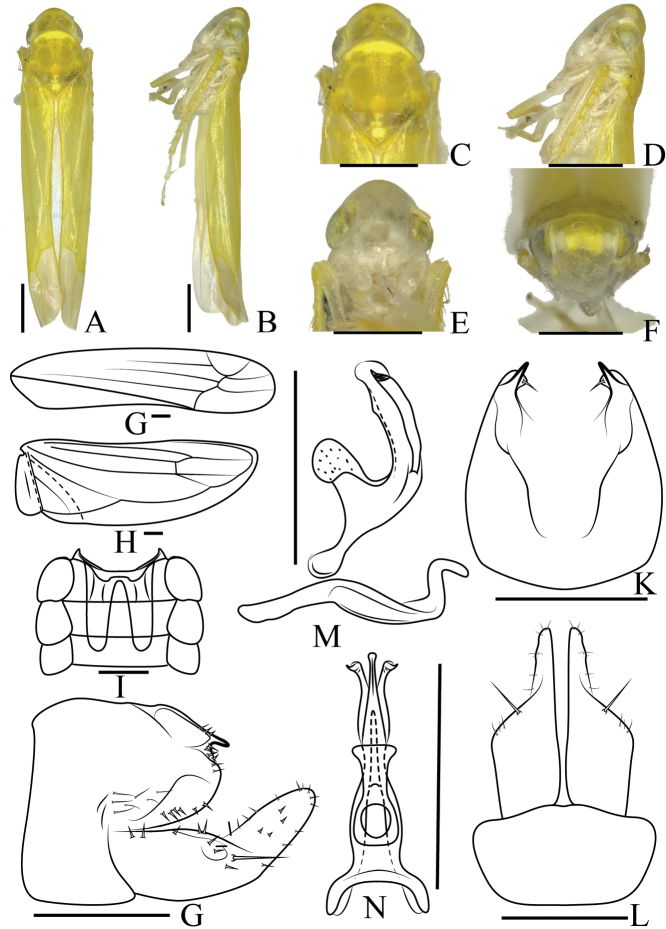
*Anakaspiralis* sp. nov. **A** male body, dorsal view **B** male body, lateral view **C** head and thorax, dorsal view **D** head and thorax, lateral view **E** face **F** head, frontal view **G** forewing **H** hindwing **I** abdominal apodeme **J** male pygofer, lateral view **K** male pygofer lobe, dorsal view **L** subgenital plate, ventral view **M** aedeagus, connective and paramere, lateral view **N** aedeagus and connective, dorsal view. Scale bars 0.5 mm (**A–F**); 0.1 mm (**G–N**).

***Length***: male 4.4–4.5 mm. ***Body*** (Fig. 5A, B) yellow. ***Crown*** (Fig. 5C) obtuse. Coronal suture distinct. ***Face*** (Fig. 5D–F) white, frontoclypeal area protuberant, anteclypeus broad. Pronotum yellow, wider than crown. ***Forewing*** (Fig. 5G) yellow with apical part white, hind wing (Fig. 5H) transparent.

***Male abdomen*** (Fig. 5I) well developed and reaching 5^th^ abdominal sternite. ***Pygofer side*** (Fig. 5J, K) broad, with small extension on superior margin, setae along periphery. Basal 1/2 of subgenital plate (Fig. 5L) broad, distal 1/2 slender in lateral view, one large macroseta at approximately mid-length. ***Paramere*** (Fig. 5M) hooked at apex. Connective fused with aedeagus. ***Aedeagus*** (Fig. 5M, N) tubular, with one pair of basal processes, apical part of processes spiral and not exceeding the stem. Gonopore apical.

#### Etymology.

The specific epithet is derived from the Latin word *spiralis* (spiraling) referring to the shape of the aedeagal processes.

#### Remarks.

In this species the aedeagus has a pair of basal processes like *A.burmensis* and *A.shashidhari*, but these basal processes have spiral-shaped top, and do not exceed the stem. These features are also not as long as in *A.nepalica*.

## Supplementary Material

XML Treatment for Anaka

XML Treatment for Anaka auricula

XML Treatment for Anaka cruciata

XML Treatment for Anaka curvata

XML Treatment for Anaka rosacea

XML Treatment for Anaka spiralis

## ﻿Additional information

### Author ORCIDs

Meng Jiao https://orcid.org/0000-0001-5081-8490

Maofa Yang https://orcid.org/0000-0002-5523-6825

Xiaofei Yu https://orcid.org/0000-0002-1362-9524

Bin Yan https://orcid.org/0000-0002-5290-4576

### Data availability

All of the data that support the findings of this study are available in the main text.
